# Supplementary material for: Alzheimer’s disease-specific cytokine secretion suppresses neuronal mitochondrial metabolism
Source: bioRxiv. 2023 Apr 7:2023.04.07.536014. Preprint. [Version 1] doi: 10.1101/2023.04.07.536014 (PMC10104145; doi:10.1101/2023.04.07.536014)
Supplement: Supplement 1 [file NIHPP2023.04.07.536014v1-supplement-1.pdf]

bioRxiv preprint doi: https://doi.org/10.1101/2023.04.07.536014; this version posted April 7, 2023. The copyright holder for this preprint (which was not certified by peer review) is the author/funder, who has granted bioRxiv a license to display the preprint in perpetuity. It is made available under aCC-BY 4.0 International license.

Supplementary Table 1: Significantly differentially expressed genes of cytokine-treated neurons compared to vehicle-treated neurons

Gene	log2FC	P-value
A2m	-0.91	0.043
Acaa2	-0.8	0.003
Acadl	0.2	0.001
Acox1	-0.44	0.004
Ak1	-0.19	0.028
Ak3	-0.37	0.040
Aldh2	-0.43	0.015
Apoe	-0.52	0.030
Ar	0.64	0.043
Atf4	0.3	0.021
Atf7ip	0.37	0.004
Atp5d	-0.26	0.010
Birc3	1.01	0.021
Brip1	1.03	0.016
Bub1	-0.47	0.007
Cacng2	-0.43	0.026
Cat	-0.23	0.014
Ccl19	1.56	0.001
Ccl2	3.44	0.001
Ccl4	-2.75	0.011
Ccl5	5.18	0.001
Ccna2	-0.68	0.009
Cd14	-2.32	0.002
Cd274	4.94	0.001
Cd36	4.36	0.000
Cdc20	-0.39	0.048
Cdk9	0.09	0.006
Cenpa	-0.56	0.015
Ctsl	-0.62	0.035
Cxcl9	5.13	0.004
Cyp1b1	-0.17	0.033
Dck	0.3	0.040
Dguok	0.34	0.020
Epc1	0.33	0.006
Fcf1	0.35	0.031
Fcgr4	2.71	0.015
Fcrls	-3.06	0.013
Fdx1	0.39	0.012
Fgf1	-1.05	0.002
Fnip1	0.18	0.031
Foxm1	-0.6	0.005
Fpr1	1.68	0.011
Gad1	-0.52	0.044
Gclc	-0.26	0.024
Glrx	0.71	0.011
Glul	-0.86	0.016
Gmpr	-0.69	0.008
Gng12	0.24	0.021
Gpx1	0.89	0.006
Gusb	-0.81	0.042
H2-Aa	9.43	0.001
H2-D1	2.74	0.019

Gene	log2FC	P-value
H2-DMa	4.32	0.000
H2-Eb1	7.61	0.001
H2-M3	2.13	0.024
H2-T23	4.42	0.014
Hacd2	-0.5	0.017
Hadh	-0.18	0.004
Hdc	1.75	0.002
Hk2	0.72	0.011
Hk3	2.25	0.014
Hsd11b1	-1.16	0.032
Hspe1	0.26	0.010
Idh3g	-0.23	0.048
Idnk	1.3	0.000
Irf1	5.15	0.000
Itch	0.18	0.030
Itga1	-0.64	0.021
Jak2	0.97	0.000
Keap1	0.51	0.002
Kif2c	-0.46	0.028
Klrk1	5.22	0.002
Kmt2a	0.41	0.050
Kyat1	-0.39	0.014
Lamc1	0.55	0.035
Lamtor2	0.13	0.001
Lck	1.24	0.020
Ldha	-0.41	0.036
Ldhb	-0.37	0.000
Map1lc3b	-0.37	0.002
Map2k2	0.21	0.010
Mki67	-0.86	0.005
Msrb2	-0.6	0.009
Mtf1	-0.16	0.032
Myb	-0.65	0.007
Myd88	1.2	0.007
Nadk	0.35	0.023
Ncoa2	0.33	0.021
Ndufa1	-0.15	0.005
Ndufa4	-0.37	0.032
Nfs1	-0.13	0.042
Nme2	0.21	0.020
Npm1	0.2	0.010
Nqo1	-1.05	0.008
Nras	0.31	0.027
Pclaf	-0.5	0.029
Pgk1	-0.38	0.017
Pik3cb	-0.26	0.040
Pik3r3	0.54	0.008
Plk1	-0.87	0.005
Pole	-0.58	0.037
Prdx5	0.38	0.001
Prim2	-0.22	0.016
Prkag1	-0.22	0.009

Gene	log2FC	P-value
Psat1	-0.34	0.030
Psma3	0.49	0.001
Psma7	0.72	0.003
Psmb10	3.27	0.004
Psmb3	0.34	0.001
Psmc1	0.14	0.014
Psmd13	0.15	0.046
Psme2	2.2	0.002
Psph	-0.16	0.015
Ptger4	-0.54	0.022
Ptges	1.22	0.001
Rgn	0.81	0.046
Rplp0	0.28	0.027
Rps6kb1	0.3	0.014
Rrm1	-0.26	0.006
Rrm2	-0.69	0.006
Scd1	-0.44	0.001
Sdhb	0.49	0.006
Sdhc	-0.05	0.012
Sec13	0.12	0.035
Slc16a1	0.19	0.027
Slc16a2	-0.46	0.001
Slc16a3	-0.5	0.001
Slc1a5	-1.14	0.002
Slc25a1	0.1	0.010
Slc7a5	-0.36	0.007
Smad4	0.17	0.022
Sox2	0.29	0.011
Sqstm1	0.31	0.001
Stat1	4.05	0.006
Stat3	0.76	0.001
Stat5a	0.46	0.020
Tbk1	0.37	0.020
Tkt	-0.2	0.022
Tlr1	0.76	0.023
Tnf	1.76	0.023
Tpr	0.22	0.025
Traf1	1.02	0.001
Traf6	0.17	0.023
Trf	-0.82	0.045
Trp53	0.4	0.016
Trp63	-0.83	0.017
Txn1	0.81	0.002
Ubb	0.38	0.003
Upp1	0.79	0.008
Uqcr11	-0.32	0.045
Usp39	0.35	0.011
Usp8	0.39	0.006
Washc4	0.4	0.020
Wrn	1.06	0.001
Zfp65	0.31	0.005
Zfp869	0.44	0.006

bioRxiv preprint doi: https://doi.org/10.1101/2023.04.07.536014; this version posted April 7, 2023. The copyright holder for this preprint (which was not certified by peer review) is the author/funder, who has granted bioRxiv a license to display the preprint in perpetuity. It is made available under aCC-BY 4.0 International license.

Supplementary Table 2: Significantly differentially expressed genes of cytokine-treated astrocytes compared to vehicle-treated astrocytes

Gene	log2FC	P-value
Ada	0.73	0.049
Akt3	0.48	0.002
Cab39	-0.22	0.025
Ccl2	2.88	0.037
Ccl5	3.55	0.016
Cd180	0.7	0.020
Cd274	5.36	0.000
Ctss	1.63	0.003
Cxcl9	3.21	0.017
Cybb	1.2	0.002
Dnajc14	-0.15	0.043
Fcgr4	2.02	0.001
Fgf1	-0.85	0.003
Gns	0.23	0.040
Gsk3b	-0.29	0.020
H2-Aa	8.65	0.001
H2-D1	5.79	0.000
H2-DMa	5.32	0.000
H2-Eb1	6.87	0.001
H2-M3	4.61	0.000
H2-T23	4	0.003
Hexa	0.27	0.003
Hk3	2.07	0.008
Hspa2	0.52	0.011
Hspa4	-0.39	0.001
Idh2	-0.49	0.036
Idnk	1.31	0.007
Irf1	4.39	0.001
Itgb2	0.91	0.041
Ldha	-0.46	0.025
Ly86	0.54	0.045
Map2k1	0.1	0.028
Mlst8	-0.39	0.026
Mycn	-0.5	0.021
Ndufa3	0.17	0.028
Pik3r1	-0.4	0.035
Prkcg	-0.63	0.032
Psma3	0.46	0.008
Psma7	0.55	0.009
Psmb10	3.73	0.000
Psmb3	0.35	0.006
Psmc1	0.17	0.013
Psmd13	0.16	0.042
Psme2	2.17	0.000
Ptk2	-0.25	0.015
Pycr1	0.59	0.021
Rbks	-0.34	0.025
Sdha	-0.25	0.050
Slc25a1	0.23	0.014
Sqstm1	0.29	0.044
Stat1	5.3	0.000
Tyms	-0.44	0.033
Washc4	0.3	0.000
Wrn	0.53	0.048
Xdh	1.67	0.006
Zfp457	-0.67	0.001
